# Development of Calorie Restriction Mimetics as Therapeutics for Obesity, Diabetes, Inflammatory and Neurodegenerative Diseases

**DOI:** 10.2174/138920210793360934

**Published:** 2010-12

**Authors:** Takuya Chiba, Tomoshi Tsuchiya, Toshimitsu Komatsu, Ryoichi Mori, Hiroko Hayashi, Isao Shimokawa

**Affiliations:** 1 Department of Investigative Pathology, Graduate School of Biomedical Sciences, Nagasaki University, 1-12-4 Sakamoto, Nagasaki 852-8523 Japan; 2 Division of Surgical Oncology, Graduate School of Biomedical Sciences, Nagasaki University, 1-7-1 Sakamoto, Nagasaki 852-8501, Japan

**Keywords:** Biosensing, calorie restriction, cancer, drug discovery, metabolism, neurodegeneration.

## Abstract

Calorie restriction (CR) is the most robust intervention that decreases morbidity and mortality, and thereby increases the lifespan of many organisms. Although the signaling pathways involved in the beneficial effects of CR are not yet fully understood. Several candidate pathways and key molecules have been identified. The effects of CR are highly conserved from lower organisms such as yeast to higher mammals such as rodents and monkeys. Recent studies have also demonstrated beneficial effects of CR in humans, although we need much longer studies to evaluate whether CR also increases the lifespan of humans. In reality, it is difficult for us to conduct CR interventions in humans because the subjects must be kept in a state of hunger and the duration of this state needed to achieve a clinically meaningful effect is still unknown. Thus, research in this field is focusing on the development of molecules that mimic the beneficial effects of CR without reducing food intake. Some of these candidate molecules include plant-derived functional chemicals (phyto-chemicals), synthetic small molecules, and endocrine molecules such as adipokines. Several studies have already shown that this research field may yield novel drugs for the treatment of age-related diseases such as diabetes. In this article, we describe the target pathways, candidate molecules, and strategies to develop CR mimetics.

## 1. INTRODUCTION 

A moderate (30–40%) restriction of calorie intake, but not malnutrition, reduces morbidity and mortality in laboratory animals compared with animals given free food access [1]. It has been shown that the anti-aging effects of calorie restriction (CR) are due to restricted dietary calorie intake, rather than the restriction of specific nutrients [2]. Compared with many other attempts to manipulate the aging process in animal models, CR remains the most robust anti-aging intervention studied to date [3]. Although, the critical signaling pathways underlying the anti-aging effects of CR have not been fully elucidated, it is thought that CR regulates the aging processes, in part, through its effects on the neural and/or endocrine regulatory systems [2]. In particular, neuropeptides and insulin/insulin-like growth factor-I (IGF-I) seem to be important molecular mediators of the adaptive metabolic response to CR [4-7]. In the neuroendocrine system, our previous study showed that the leptin–neuropeptide Y (NPY) pathway has important roles in the effects of CR [8, 9]. Because of the robustness of CR, many researchers have tried to identify the molecules that mimic the beneficial effects of CR as candidate drugs for age-related diseases [3, 10]. Some of these results suggest that this research field could be a promising approach to develop drugs for human diseases such as obesity, diabetes, inflammatory and neurodegenerative diseases [11].

## 2. EFFECTS OF CALORIE RESTRICTION IN HUMANS 

A longitudinal epidemiological study has been conducted in Baltimore (ML, USA, Baltimore Longitudinal Study of Aging), and many interesting findings have reported. One of the most remarkable results among those studies is the finding of biomarkers of longevity in humans. The study showed that low body temperature, low blood insulin, and high de-hydroepiandrosterone sulfate were characteristics of human longevity [12]. Those changes were similar to those observed in CR rodents and monkeys. Although these are indirect findings, the results suggest that CR in humans might also have beneficial effects, as observed in laboratory animals. On the other hand, several ongoing projects have subjected humans to CR directly. One of these studies, the CALERIE (Comprehensive Assessment of Long-term Effects of Reducing Intake of Energy) project, has started in multiple institutions in the United States, and some positive effects on human health of 6 months of CR have already been reported [13]. However, the complexity of human genetics and the relatively short duration of these projects, as compared with the human lifespan, make it difficult to evaluate the long-term effects of CR on humans. Clearly, a life-long clinical study is needed to confirm the anti-aging effects of CR in humans, although this is unrealistic. Even if science has advanced sufficiently and can clearly show that CR in humans can reduce morbidity and mortality, and maintain function to the same degree as observed in experimental animals, questions remain as to whether to pursue CR in humans. First, we must be kept in hunger for a significant period of time during daily life. Second, we do not yet know how great and long the CR needs to be in humans to achieve clinically relevant effects. Third, CR in humans could have adverse effects such as infertility and osteoporosis. These obstacles have encouraged researchers to identify and develop molecules that can mimic the anti-aging, pro-longevity effects of CR, without reducing caloric intake.

## 3. CR MIMETICS 

The concept of CR mimetics is relatively new [3]. It has been proposed that CR mimetics should have the following key features: (i) they should mimic the metabolic, hormonal and physiological effects of CR; (ii) they should not significantly decrease long-term food intake; (iii) they should activate stress–response pathways, as observed in CR, and protect against a variety of stressors; and (iv) they should reduce inflammation and autoimmunity. Several studies have been conducted to evaluate whether existing drugs mimic these properties using microarray-based gene expression analysis in mice [14, 15]. The results of those studies suggest that the anti-diabetic drug metformin and peroxisome proliferator-activated receptor α (PPARα) activators are potential candidate CR mimetics. The results also suggest that modulation of metabolism (including glucose and lipid metabolism) and inflammation could be major targets for the development of CR mimetics in terms of similar gene expression patterns. Therefore, CR mimetics could be used for the treatment of obesity, diabetes, and inflammatory diseases.

### 3.1. Metformin 

Metformin, one of the most widely used type 2 diabetes therapeutics, requires liver kinase B1 (LKB1) in the liver to reduce blood glucose levels [16]. LKB1 phosphorylates and activates AMPK [adenosine monophosphate (AMP)-activated protein kinase]. Deletion of LKB1 in the liver of mice resulted in an almost complete loss of AMPK activity. Furthermore, the loss of LKB1 function resulted in hyperglycemia with increased gluconeogenic and lipogenic gene expression. We previously found that AMPK activity was decreased in the liver of CR rats [17]. We also found enhanced expression of gluconeogenic and lipogenic genes in the CR rat liver [8, 18]. These results suggest that, at least in the liver, AMPK activity should be downregulated to maintain glucose and lipid metabolism in CR rats. Even though metformin can activate AMPK, it is a potential CR mimetic [15]. Interestingly, their microarray study revealed that 8 weeks of metformin treatment had more pronounced effects on CR-like gene expression than 8 weeks of CR. Thus, in the liver, metformin might optimize the activity or responses of the LKB1–AMPK pathway to maintain appropriate glucose and lipid metabolism, depending on the energy requirements. We also found that a novel insulin signaling molecule, WD-repeat protein 6 (WDR6), interacts with LKB1 [19-21]. Therefore, this molecule might also be involved in the adaptation of glucose and lipid metabolism in CR, and is a putative target for the development of CR mimetics. 

Metformin treatment in cancer-prone female human epidermal growth factor receptor type 2 (HER-2)/neu transgenic mice induced a phenotype similar to that in CR mice, with suppression of the age-associated rise in blood glucose and lipid levels [22]. In that study, metformin significantly decreased the incidence and size of tumors, and increased the lifespan of mice [22]. However, metformin did not significantly increase the lifespan of normal Fischer 344 rats compared with control rats [23]. These results suggest that the CR-mimetic effects of metformin are species-specific or function only in disease-prone conditions. Nevertheless, these results suggest that modulation of the LKB1–AMPK signaling pathway could be an attractive target for the development of CR mimetics. 

### 3.2. Adipokines

Adipokines, adipose tissue-derived hormonal factors, could be important mediators of the beneficial effects of CR [5]. We previously demonstrated that downregulation of leptin and resistin, and upregulation of adiponectin are characteristics of CR animals [8, 24, 25]. Adiponectin is a positive regulator of the beneficial effects of CR and may be an important target for the development of CR mimetics. Yamauchi *et al. *[26] showed that adiponectin has two different receptors, adiponectin receptors 1 and 2 (AdipoR1 and AdipoR2), which have important roles in the regulation of glucose and lipid metabolism, inflammation and oxidative stress *in vivo*. In the liver, AdipoR1 activates the AMPK pathways and AdipoR2 activates PPARα pathways [26]. A recent study showed that AMPK controls the expression of genes involved in energy metabolism in mouse skeletal muscle by acting in coordination with SIRT1 (sirtuin 1) [27], an NAD^+^-dependent deacetylase [28]. AMPK enhances SIRT1 activity by increasing cellular NAD^+^ levels, resulting in the deacetylation and modulation of the activity of downstream SIRT1 targets such as PPARγ coactivator 1α (PGC-1α) and forkhead box O1 (FoxO1). Moreover, this AMPK–SIRT1 pathway in muscle is regulated by the adiponectin–adipoR1 system [29]. Taken together with the finding that CR does not activate AMPK [17] or SIRT1 [30] in the liver, CR mediated upregulation of adiponectin might contribute to the regulation of metabolism in muscle, but not in the liver. Therefore, development of CR mimetics that target the adiponectin–AMPK pathway should be activated in a tissue-specific manner.

### 3.3. NPY

We previously reported that NPY has important roles in the neuroendocrine adaptations of CR [8-9]. NPY was found to enhance the stress response and regulate the expression of adipokines [8]. Moreover, NPY transgenic rats showed an increased lifespan [31]. One of the major endogenous NPY activators is the gut-derived hormone ghrelin [32, 33]. In both rodents and humans, plasma ghrelin concentrations increase during fasting to stimulate the feeding behavior. CR can suppress the growth hormone (GH)–IGF-I pathway, but ghrelin activates pituitary GH secretion *via *its receptor, GHS-R. Therefore, during CR, the increased expression of ghrelin might selectively activate the NPY pathway but not GH secretion. Indeed, we found an inverse relationship between NPY and GH gene expression in CR rats [8]. It has been shown that infusion of thyroid hormone [triiodothyronine (T3)] activated the hypothalamic NPY–NPY-Y1 receptor pathway by reducing the circulating leptin level, but not by increasing the circulating ghrelin level [34]. Therefore, leptin signaling might be more sensitive than ghrelin signaling to induce NPY expression in the hypothalamus. Interestingly, a recent study showed that SIRT1 regulates thyroid-stimulating hormone release from pituitary cells, which is involved in T3 secretion [35]. These results suggest that sirtuin activators might have potential effect as NPY activators by modulating T3 induction to reduce the leptin level and thereby increase NPY activity. 

### 3.4. Rapamycin

Inhibition of the target of rapamycin (TOR) signaling pathway by genetic or pharmacological intervention extends lifespan in yeast, nematodes and flies [36-40]. In these three organisms, CR is mediated, at least in part, by reduced TOR signaling [37, 38, 41, 42]. Moreover, mice fed with rapamycin to inhibit the mTOR pathway showed significantly increased lifespan compared with control mice [43]. They also evaluated the phosphorylation levels of ribosomal protein subunit S6 (rpS6), a target substrate of S6 kinase 1 (S6K) in the mTOR signaling pathway [44]. Rapamycin-fed mice showed a significant reduction in the levels of phosphorylated rpS6 compared with control mice in adipose tissue. Consistent with those findings, knockout of S6K increased the lifespan of mice [45]. Interestingly, sestrin was identified as a feedback inhibitor of TOR in flies [46]. Loss of sestrin resulted in age-associated pathologies including triglyceride accumulation, mitochondrial dysfunction, muscle degeneration and cardiac malfunction, which were prevented by activation of AMPK or inhibition of TOR. These results indicate that the TOR pathway could be an important signaling pathway that regulates various biological functions implicated in aging [47]. Therefore, rapamycin and related molecules that inhibit the TOR pathway may be attractive candidate CR mimetics.

### 3.5. Sirtuin Activators

#### 3.5.1. Resveratrol

Orthologs of mammalian SIRT1 (Sir2 in yeast, sir-2.1 in nematodes, dSir2 in flies) were shown to promote longevity in these species in some conditions [48]. Because CR did not extend the lifespan of SIRT1-deleted organisms, they considered that the beneficial effects of SIRT1 activation and CR involved similar pathways [48]. Howitz *et al. *[49] performed a screening study to identify activators of human SIRT1 and reported several candidate molecules, which included resveratrol. Resveratrol is a polyphenolic compound present in high amounts in red grapes and in wine [50]. Resveratrol has been shown to increase the lifespan of nematodes and fruit flies in a sirtuin-dependent manner, which appeared to mimic CR [51]. Furthermore, epidemiological studies have demonstrated that consumption of resveratrol could reduce the risk of many age-related diseases [52]. However, other reports have suggested that resveratrol does not have pro-longevity effects in yeast [53]. Moreover, the pro-longevity effects of resveratrol in nematodes and fruit flies were not observed in another study [54]. Meanwhile, one study showed that resveratrol inhibited SIR-2.1 activity in nematodes [55].

On the other hand, in a study in mice, resveratrol shifted the physiology of middle-aged mice fed with a high-calorie diet towards that of control diet-fed mice, and significantly increased their lifespan [56]. Resveratrol induces various changes, as observed in CR mice, including increased insulin sensitivity, reduced IGF-I levels, and increased PGC-1α activity [56]. In other studies, resveratrol was shown to influence gene expression in multiple tissues, with similar changes to those induced by CR. However, mice fed a standard diet did not live longer when they were treated with resveratrol starting at 12 months of age [50]. These results suggest that the CR-mimetic effects of resveratrol are more evident in disease states, such as obesity and diabetes in mice. 

#### 3.5.2. Other Small Molecules

Milne *et al. *[57] published results of their study in which they identified extremely strong SIRT1 activators from a small molecule library. One of the molecules, SRT1720, is structurally unrelated to, but was 1000-fold more potent than resveratrol for the activation of SIRT1 in their assay system. Using obese mice, they also showed that SRT1720 improved insulin sensitivity, lowered blood glucose, and increased mitochondrial capacity [57]. These results suggest that SIRT1 activators offer a promising new approach to treat age-related diseases such as diabetes, and this compound is currently being tested in clinical trials [57]. However, a more recent study demonstrated that resveratrol and SRT1720 do not directly activate SIRT1 [58]. In fact, they reported a potential problem with the SIRT1 activator screening system used to identify resveratrol and SRT1720, as described below.

## 4. SCREENING OF CR MIMETICS

Pacholec *et al. *[58] assessed the previously identified SIRT1 activators using biochemical assays with native substrates, including a p53-derived peptide substrate without a fluorophore, as well as the purified native full-length protein substrate p53. They found that resveratrol and SRT1720 did not activate SIRT1 with the native peptide or full-length protein substrates. These compounds only activated SIRT1 with a peptide substrate containing a covalently attached fluorophore, as observed in an earlier study that identified several SIRT1 activators [57]. Therefore, they concluded that resveratrol and SRT1720 are not direct activators of SIRT1. They also revealed that there are various off-targets of these sirtuin activators, which showed no beneficial effects in high-fat diet-fed mice. Howitz *et al. *[49] originally developed an *in vitro* screening system for CR mimetic sirtuin activators. However, it has already been reported that this screening system may be affected by the use of fluorophores [53]. It is possible that fluorophores might mimic an unidentified protein function needed for *in vivo* activation of SIRT1 by these compounds. In fact, the beneficial effects of these molecules have not reproduced *in vivo *[58]. Most recently, Dai *et al. *[59] reported that some sirtuin activators could accelerate SIRT1-catalyzed deacetylation of unlabeled peptides, and that the activators interact directly with SIRT1 and activate SIRT1 activity through an allosteric mechanism. However, they would need to show that their compounds enhance deacetylation of native p53 to confirm their conclusions. Therefore, we believe that further studies are needed to resolve this controversy, although SIRT1 activation in specific tissues might offer a promising approach to develop CR mimetics. On the other hand, novel screening systems are needed to identify possible candidate CR mimetics. 

The development of an effective screening system will greatly accelerate the identification of potential candidates and expand this research field. Screening processes can be based on cell systems or animal model systems, such as biosensor mice using a secreted alkaline phosphatase reporter [60, 61]. To do this, we must identify the sequence of the *cis*-acting elements upstream of the potential pro-longevity genes. The accumulation of DNA microarray data would be helpful to achieve this goal. Such approaches have already been used to identify various regulatory systems in yeast, and have identified several sequence motifs, such as those that are important in the heat shock stress response [62]. Such strategies could be exploited to develop CR mimetic screening systems using the previously reported data [63]. We have developed such a screening system, which we have named as CRISP—the CR-Imitating agent Screening Platform [64]. We have already demonstrated the effectiveness and specificity of this screening platform. Nevertheless, candidate CR mimetics must still be verified by showing that their beneficial effects are similar to those of CR. Such compounds should function in healthy animals and in animal models of disease, without any deleterious effects. This stage of development would be the most time- and cost-consuming process. Moreover, large-scale collaboration is necessary to confirm the effects of candidate molecules, such as that conducted in the assessment of rapamycin, which showed pro-longevity effects in mice bred at different institutions [43]. 

## 5. CONCLUSIONS

There are multiple *in vivo* targets involved in the effects of CR. Therefore, it is likely that there are other pathways that regulate aging and age-related disorders, to mediate the effects of CR, other than those discussed in this article. Accordingly, approaches involving a single molecule or a single pathway might show limited effects as a CR mimetic. However, the development of CR mimetics may offer an elixir for healthy life of humans, and recent and ongoing studies are showing much promise to accomplish this goal. This field of research has attracted scientists from many different backgrounds, including neurobiologists, endocrinologists, nutritional scientists and biomedical gerontologists. Interdisciplinary and integrated collaboration of these disciplines would accelerate the research endeavors.
